# Asynchronous c-VEP communication tools—efficiency comparison of low-target, multi-target and dictionary-assisted BCI spellers

**DOI:** 10.1038/s41598-020-74143-4

**Published:** 2020-10-13

**Authors:** Felix W. Gembler, Mihaly Benda, Aya Rezeika, Piotr R. Stawicki, Ivan Volosyak

**Affiliations:** Rhine-Waal University of Applied Sciences, Technology and Bionics, 47533 Kleve, Germany

**Keywords:** Neuroscience, Medical research, Engineering

## Abstract

Keyboards and smartphones allow users to express their thoughts freely via manual control. Hands-free communication can be realized with brain–computer interfaces (BCIs) based on code-modulated visual evoked potentials (c-VEPs). Various variations of such spellers have been developed: Low-target systems, multi-target systems and systems with dictionary support. In general, it is not clear which kinds of systems are optimal in terms of reliability, speed, cognitive load, and visual load. The presented study investigates the feasibility of different speller variations. 58 users tested a 4-target speller and a 32-target speller with and without dictionary functionality. For classification, multiple individualized spatial filters were generated via canonical correlation analysis (CCA). We used an asynchronous implementation allowing non-control state, thus aiming for high accuracy rather than speed. All users were able to control the tested spellers. Interestingly, no significant differences in accuracy were found: 94.4%, 95.5% and 94.0% for 4-target spelling, 32-target spelling, and dictionary-assisted 32-target spelling. The mean ITRs were highest for the 32-target interface: 45.2, 96.9 and 88.9 bit/min. The output speed in characters per minute, was highest in dictionary-assisted spelling: 8.2, 19.5 and 31.6 characters/min. According to questionnaire results, 86% of the participants preferred the 32-target speller over the 4-target speller.

## Introduction

Brain–computer interfaces (BCIs) hold the potential to aid people with severe clinical disorders in their daily life as they allow hands-free control and communication. These systems translate the BCI users’ brain activity, usually acquired non-invasively via electroencephalography (EEG), into control commands for external devices^1^. For example, BCIs may serve as communication tools for people who cannot use typical manual input devices.

Various BCI communication applications (typically referred to as spellers) have been realized over the last years. They have been categorized according to the analysed brain signal (e. g. event-related potentials or sensorymotor rhythms), the graphical user interface (GUI) design (multi-step versus single-step), the selection interval mechanism (synchronous versus asynchronous) and the usage of additional features (e. g. word completion methods)^2^.

Brain signals used for BCI control also include visual evoked potentials (VEPs) which have been studied since the 1970s^3^. Nowadays, two VEP approaches are predominantly used in BCI research: the frequency-modulated VEPs (f-VEPs)^4–7^ and the code-modulated VEPs (c-VEPs)^8–11^. In spellers based on VEPs, several stimuli classes, each flickering with a unique pattern, represent control commands; for example, for selecting letters of a virtual keyboard. The BCI classifies which target the user is looking at by interpreting the brain signals in real-time. For the c-VEP paradigm (used in the presented study), the flickering patterns are modulated with different time lags of a binary code sequence; EEG templates for each stimulus class need to be generated from data collected in a recording session.

BCI spellers can employ a low-target (multi-step) or a multi-target (single-step) graphical user interface (GUI) design. In low-target interfaces, several selections are needed to choose the desired character. A low number of stimulus classes are sufficient; spellers with only four or five different flickering patterns are quite common^4,12–15^. While low-target spellers allow high classification accuracies, the overall spelling speed is limited, as several selection intervals (typically consisting of stimulation intervals and flicker-free intervals for gaze-shifting, where users can shift their gaze to the next target) are required for letter selections.

Multi-target spellers, on the other hand, employ a single-step GUI design and typically resemble a QWERTY-style keyboard layout^16–19^ or use an alpha-numeric letter arrangement^7,10,11,20^. These interfaces usually use 28^19^ to 55^17^ stimulus targets to present the English alphabet consisting of 26 letters and sometimes additional characters such as numbers or punctuation marks. According to recent publications, the highest spelling speeds were achieved with an alpha-numeric 40-target interface developed by Chen et al.^7^, who reported an average information transfer rate (ITR) of 267 bit/min employing 0.5 s stimulation intervals and 0.5 s gaze-shifting intervals. Employing even shorter stimulation time windows of 0.3 s, Nakanishi et al.^21^ reported average ITRs of 325 bit/min (cue-guided selection task) and 199 bit/min (copy-spelling task).

While multi-target spellers allow faster speeds, they may cause more eye fatigue than low-target spellers, as was observed in SSVEP studies (e. g.^22^). More importantly, due to the high number of targets, which need to be distiguished by the system, these systems tend to be less precise. Bin et al.^23^ tested both a 16-target and a 32-target c-VEP system and observed that doubling the number of targets caused an accuracy drop from 92% to 85%. Moreover, so-called BCI illiteracy cases, where users were not able to achieve sufficient control over multi-target systems, have been reported repeatedly^11,19,24^. For example, Renton et al.^19^ reported that almost half of 38 participants did not achieve sufficient accuracies for reliable free communication with a 28-target f-VEP speller employing 1.5 s stimulation intervals (i. e. <80% accuracy in their preliminary assessment).

Many researchers focus on improving classification accuracy to reduce BCI illiteracy. The classical classification method involving canonical correlation analysis (CCA)^5,25^ has been improved several times. Chen et al.^20^ suggested CCA classification based on filter banks for the f-VEP paradigm. Their method decomposes the original data by applying several band-pass filters. The authors tested several different decomposition designs: equally spaced, harmonic and overlapping sub-bands, and observed that the latter yielded the highest accuracy. Recently, Monidini et al.^26^ investigated the number of correlations coefficients considered for CCA classification and found a significant improvement in classification accuracy if more than one coefficients (as in the conventional approach) were considered.

An approach to improve both the classification accuracy and the overall system usability is a dynamic classification window paradigm. The stimulation intervals can either be determined by the system (synchronous spellers^7,11,23^) or involve on-line classification scores based on real-time EEG data, which are compared to threshold values (asynchronous spellers^12,17,27^). The latter approach reduces unintended selections (often referred to as the Midas touch problem^28^) and significantly increases accuracies in practical spelling scenarios. While synchronous applications with small stimulation intervals are often tested to demonstrate high ITRs, they may overestimate the true communication speed achievable in a realistic setting. On the other hand, although generally slower, asynchronous applications may achieve a more naturalistic communication and are better suited for naive users.

A way to improve spelling efficiency for asynchronous spellers is word completion features, which allow users to spell words with fewer selections. While several word prediction features have been implemented for other BCI paradigms such as event-related potentials^29,30^, they are rarely used in BCIs based on VEPs. The few VEP spellers with dictionary support fall in the category of asynchronous low-target systems: Volosyak et al.^31^ presented a dictionary feature for an asynchronous multi-step f-VEP system (the so-called Bremen-BCI speller), where a drop-down list containing six dictionary suggestions was employed; more recently, we presented an asynchronous multi-step 8-target c-VEP system offering word suggestions based on an n-gram word prediction model^32^. For multi-target systems, these kinds of features may be beneficial as well.

To investigate what kind of speller (multi-step, single-step, dictionary-assisted) is ideal in terms of reliability, speed, cognitive load, and visual load, we tested a 4-target system (low-target, multi-step) and a 32-target (multi-target, single-step) system using the c-VEP paradigm. The latter system also offered dictionary suggestions. A large subject group (58 participants) went through different spelling tasks: letter-by-letter spelling tasks to investigate the effect of the numbers of targets and dictionary-assisted spelling to investigate the efficiency of the word prediction feature.

For signal classification, we used a new ensemble approach employing multiple spatial filters based on CCA correlation coefficients. Moreover, as naturally occurring EEG activity (e. g. alpha activity when closing the eyes) may lead to false classifications, the original EEG data were decomposed into alpha-band (8–12 Hz), beta-band (approx. 12–30 Hz), and gamma-band (>30 Hz) related activities. Weights for this filter bank design were determined individually based on the training data. The idea behind the approach was to enhance the separation between natural brain activity and stimuli induced responses. For example, for some users, natural alpha activity (associated with tiredness) may interfere with cVEP detection. Due to the individual weights the impact of alpha activity on classification can be reduced in such cases.

In summary, the overall aims of the study were the following:

- Confirming our previous results that all subjects are able to use c-VEP-based BCIs,
- Comparing low-target and multi-target BCIs in terms of user-friendliness, accuracy and speed,
- Evaluating the efficiency of dictionary features for asynchronous multi-target BCIs,
- Evaluating the proposed classification model based on individualized filter bank design and multiple spatial filters.

Next to the typical performance metrics employed in BCI research, we assessed subjective opinions after training and spelling phases using multiple questionnaires.

## Results

The overall performance of the spellers was assessed via classification accuracy, selection time (including 1 s gaze shift), ITR^1^, and output characters per minute (OCM)^29^ (see Methods section). The results of the off-line cross-validation and the on-line spelling experiments for the 4-target and 32-target system are presented in the following.

### On-line spelling phase

**Figure 1:**
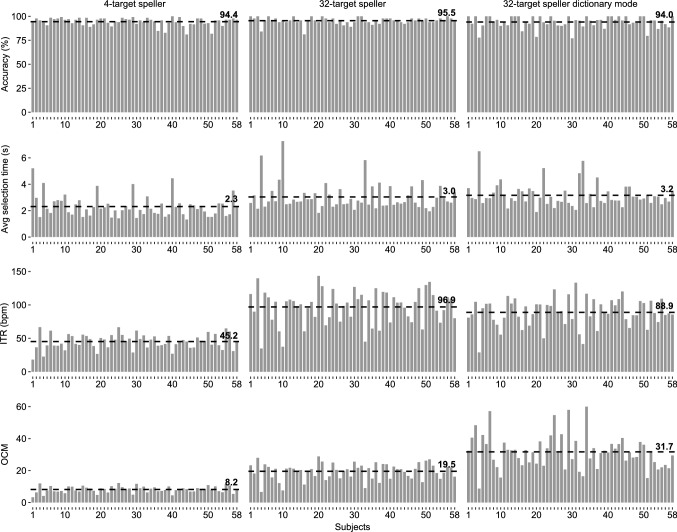
Results of the on-line spelling tasks. For each participant, the classification accuracy, average selection time, information transfer rate (ITR), and output characters per minute (OCM) after letter-by-letter spelling with the 4-target speller, letter-by-letter spelling with the 32-target speller and dictionary-assisted spelling with the 32-target speller are presented. The letter-by-letter spelling task was the same for both systems (the pangram “THE QUICK BROWN FOX JUMPS OVER THE LAZY DOG”). The dictionary-assisted spelling task was an English sentence (for each participant, an individual sentence ranging from 24 to 51 characters). The dashed lines indicate the means across participants.

Three spelling tasks were performed: letter-by-letter spelling with the 4-target GUI, letter-by-letter spelling with the 32-target GUI (in both cases the same pangram was spelled), and dictionary-aided spelling with the 32-target GUI (different real world English sentences were spelled). All 58 participants successfully completed the three spelling tasks (BCI literacy rate 100%); Figure 1 displays the individual results. In the following, mean (M) scores and standard deviations (SDs) are listed. Paired two-tailed Students *t*-tests were used to investigate differences in performance between the 32- and 4-target spellers, and between the dictionary-supported spelling and standard letter-by-letter spelling.

Regarding the differences between 32 and 4 targets in on-line performance, the letter-by-letter spelling tasks were performed with high accuracies for the 32-target system ($M=95.5\%$, $SD=3.6$) and for the 4-target system ($M=94.4\%$, $SD=4.3$) system. The difference in accuracy was not significant ($t=1.42$, $p=0.16$). The mean selection time was longer for the 32-target system ($M=3.04$ s, $SD=1.01$) than for the 4-target system ($M=2.31$ s, $SD=0.80$). This difference was significant $t=5.60$, $p<0.0001$. The mean ITR was higher for the 32-target system ($M=96.9$ bit/min, $SD=24.9$) than for the 4-target system ($M=45.2$ bit/min, $SD=10.9$). This difference was significant $t=17.2$, $p<0.0001$. Similarly, the mean OCM in letter-by-letter spelling was significantly higher ($t=19.1$, $p<0.0001$) for the 32-target system ($M=19.5$ characters/min, $SD=5.0$) than for the 4-target system ($M=8.2$ characters/min, $SD=2.0$).

To investigate the differences between dictionary-supported spelling and standard letter-by-letter spelling, the on-line performances of the respective tasks with the 32-target system were evaluated. High mean accuracies in letter-by-letter spelling ($M=95.5\%$, $SD=3.6$) and dictionary-supported spelling ($M=94.0\%$, $SD=6.0$) were achieved. The difference in accuracy was not significant ($t=1.9$, $p=0.06$). The mean selection time was slightly shorter in letter-by-letter spelling ($M=3.04$ s, $SD=1.01$) than in dictionary-supported spelling ($M=3.16$ s, $SD=0.87$). This difference was also not significant $t=1.32$, $p=0.19$. The mean ITR was slightly higher in letter-by-letter spelling ($M=96.9$ bit/min, $SD=24.9$) than in dictionary-supported spelling ($M=88.9$ bit/min, $SD=20.8$). This difference was significant $t=2.6$, $p=0.01$. On the other hand, due to the dictionary suggestions, the mean OCM was significantly higher ($t=9.1$, $p<0.0001$) in dictionary-supported spelling ($M=31.7$ characters/min, $SD=10.5$) than in letter-by-letter spelling ($M=19.5$ characters/min, $SD=5.0$).

Additional exploratory analysis was conducted to investigate differences between male and female participants. Welch’s two sample *t*-tests were conducted for the accuracies reached with the 4-target and 32-target letter-by-letter tasks. For the 4-target speller, the difference between female ($M=95.6\%$, $SD=2.6$) and male ($M=93.3\%$, $SD=5.3$) participants was significant ($t=2.1$, $p=0.04$). In the same way, for the 32-target speller, the difference between female ($M=96.6\%, SD=2.5$) and male ($M=94.3\%$, $SD=4.2$) participants was significant ($t=2.5$, $p=0.02$).

### Questionnaire results

After off-line recording sessions and spelling sessions, participants went through questionnaires (for more details refer to the experimental procedure in the Methods sections).

A series of two-tailed Wilcoxon signed-rank tests were conducted to examine differences regarding the subjective user impressions between the 4-target and the 32-target speller. In the following, median scores, interquartile ranges (IQRs) and ranges are listed.

**Figure 2:**
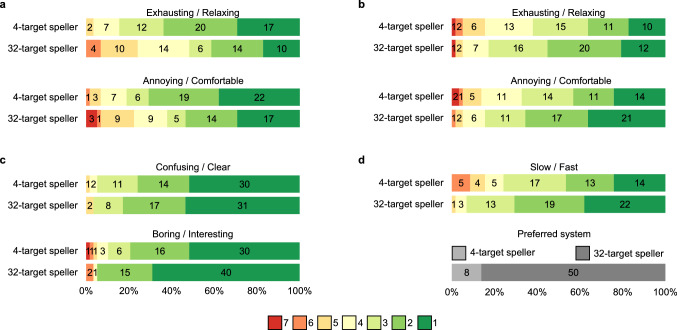
Questionnaire responses of 58 participants to statements regarding training session and spelling interface. Answers were given on a 1–7 Likert scale. The numbers in the bars indicate the absolute numbers of participants that gave the respective rating. (**a**) Post-training (prior to the on-line experiments) questionnaire related to the stimulus presentation. (**b**) Post-spelling questionnaire related to the stimulus presentation. (**c**) Post-spelling questionnaire related to the GUI design. (**d**) Post-spelling questionnaire related to the overall BCI performance.

Figure 2a shows the post-training questionnaire results related to the flickering. For the Likert item exhausting (7)/relaxing (1), the median score for the 4-target speller was 2 (IQR 2, range 1–5) compared to 3 (IQR 2, range 1–6) for the 32-target speller. For the Likert item annoying (7)/comfortable (1), the median score for the 4-target speller was 2 (IQR 2, range 1–6) compared to 2 (IQR 3, range 1–7) for the 32-target speller. In both cases, answers for the 4-target speller were shifted more towards the positive statement, i.e. the lower number ($p<0.001$).

Figure 2b shows the post-spelling questionnaire results related to the flickering. For the Likert item exhausting (7)/relaxing (1), the median score for the 4-target speller was 3 (IQR 2, range 1–7) compared to 2 (IQR 2, range 1–7) for the 32-target speller. For the Likert item annoying (7)/comfortable (1), the median score for the 4-target speller was 3 (IQR 2, range 1–7) compared to 2 (IQR 2, range 1–6) for the 32-target speller. Now, in both cases, answers for the 32-target speller were shifted more towards the positive statement ($p<0.001$).

Figure 2c shows the post-spelling questionnaire results related to the GUI. For the Likert item confusing (7)/clear (1), the median score for the 4-target speller was 1 (IQR 1, range 1–5) compared to 1 (IQR 1, range 1–5) for the 32-target speller. The question did not result in a significant difference ($p=0.57$). For the Likert item boring (7)/interesting (1), the median score for the 4-target speller was 1 (IQR 1, range 1–7) compared to 1 (IQR 1, range 1–6) for the 32-target speller. For this Item, the answers for the 32-target speller were shifted more towards the positive statement ($p=0.001$).

Figure 2d shows the post-spelling questionnaire results related to the performance. For the Likert item slow (7)/fast (1), the median score for the 4-target speller was 3 (IQR 1, range 1–6) compared to 2 (IQR 2, range 1–5) for the 32-target speller. The answers for the 32-target speller were shifted more towards the positive statement ($p<0.001$). Finally, when asked, which of the two systems they preferred, 50 participants (86.2%) voted for the 32-target speller.

### Off-line results

The recorded training data was used for exploratory analysis via a 4-fold stratified cross-validation^33^. The results were then averaged across folds.

**Figure 3:**
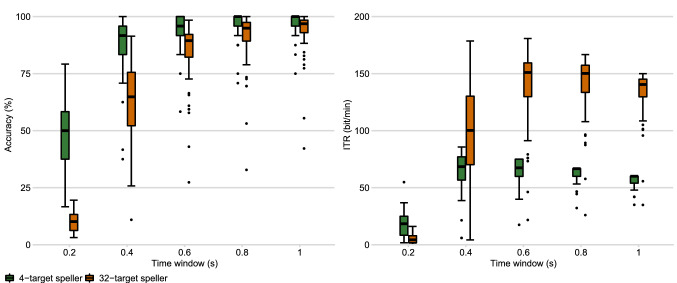
Off-line comparison of the BCI performance of the 4-target and the 32-target speller. The dipicited box plots show the distribution of off-line accuries and ITRs across the 58 participants at different time windows. The boxes indicate the middle 50%, the lines that devide the boxes indicate the median, the antennas indicate the upper and lower quartile, and the individual points indicate outliers, i. e. data points outside 1.5 times the interquartile range.

Figure 3 shows the accuracies and ITRs for time windows up to 1 s. As expected, the accuracies are considerably higher for the 4-target system, while ITRs are considerably higher for the 32-target system.

**Figure 4:**
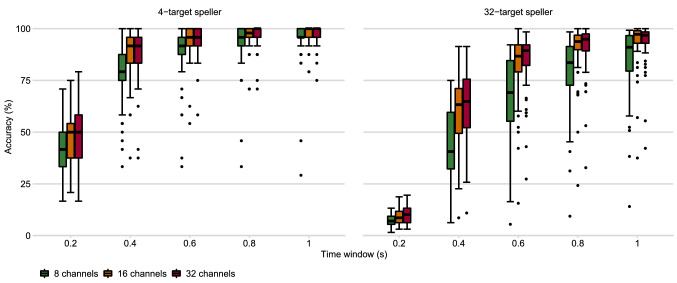
Classification accuracies for different electrode configurations. Off-line accuracies across the 58 participants calculated for 8, 16 and 32 channels are provided at different time windows.

To further compare 4-target and 32-target systems, the effect of the number of electrodes on BCI performance was analysed. Figure 4 shows classification accuracies for the 4-target and the 32-target speller for different channel montages around the visual cortex. The classification accuracy decreases with the reduction of electrodes. The drop in accuracy from 16 to 8 electrodes is much larger than that from 32 to 16 electrodes.

**Figure 5:**
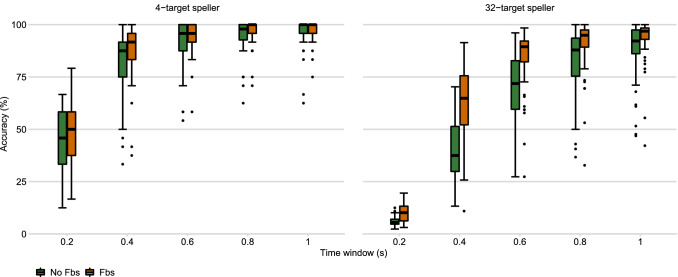
Off-line-comparison of the BCI performance using the standard classification and filter bank approach for the 4-target and the 32-target speller.

The effectiveness of the proposed classification (filter bank approach and adaptive weight mechanism) was assessed. Figure 5 compares the off-line accuracies of the standard c-VEP classification method (i. e. without filter bank decomposition) and the proposed method. The median accuracies were generally higher with the suggested methods. However, for the 4-target speller, only low classification time windows yielded considerable differences.

## Discussion

For practical BCI applications classification accuracy, communication speed, and robust non-control state are essential. The latter point is particularly crucial, as in true communication, users do not always intend to enter commands for certain time periods. In asynchronous implementations, output commands are only produced if the user intends to do so.

One aim of the study was to explore the efficiency of the asynchronous dictionary-supported multi-target c-VEP system. We used a dynamic time window mechanism employing a threshold-based classification approach. For the user, this means the flicker intervals changed dynamically, the flicker-free gaze-shifting phases were set to 1 s in this study. Various other studies employ shorter gaze-shifting phases of 0.5 or 0.75 s. In preliminary tests, we found that such short intervals may reduce classification accuracy, especially for users who are unfamiliar with the system. This is inline with remarks by Chen et al., who used 1 s gaze shifting windows to increase accuracy in some cases.

Several other asynchronous VEP spellers have been developed previously^4,17,32,34^: For example, Cecotti et al.^12^ achieved 37.6 bit/min and 5.5 characters/min with 8 participants testing a 4-target menu-based f-VEP speller. Volosyak et al.^4^ achieved 61.7 bit/min and about 10 characters/min with 7 participants testing a 5-target letter grid layout employing the f-VEP paradigm. In a previous study^32^, we tested an 8-target layout with 18 participants using n-gram dictionary functionality and achieved an ITR of 57.8 bit/min and 18.4 characters/min with different English sentences. Nagel et al.^17^ reported 109.1 bit/min and 16.1 characters/min with 10 participants who used a 55-target German QWERTZ-layout, spelling 3 times the German phrase “Asynchron BCI” (case sensitive). In terms of ITR, these results were among the fastest reported for asynchronous applications. For the 32-target speller used in this study, slightly lower ITRs of about 90 bit/min were achieved; but due to the dictionary integration, the average output character speed increased to 31.6 characters/min on the average (up to 60 characters/min) in dictionary-assisted spelling. Notably, despite the high number of participants and the complexity of the spelling tasks, this is the highest character output efficiency reported with asynchronous applications until now. We would like to point out that the reported OCM values are highly dependent on the complexity of the sentence tasks. For simple sentences, dictionary suggestions are more helpful resulting in higher OCM scores. For example, S20 and S21 both achieved an ITR of roughly 100 bit/min; the OCMs, however, were quite different (S20 achieved 24.2 spelling “LIBERTY CONSISTS IN DOING WHAT ONE DESIRES” and S21 achieved 38.2 characters/min spelling “I WILL TRY TO MAKE IT RIGHT THIS TIME”). The ITR in dictionary-assisted spelling was slightly lower than in the pangram spelling task (88.9 bit/min versus 96.9 bit/min). The reduced ITR can be attributed to additional search phases and increased mental load. The fact that accuracy remained high (no significant difference) demonstrates the robustness of the asynchronous selection paradigm. It should be highlighted that subjects used the GUI for the first time. The dictionary function was not used optimal by the participants. In some cases, useful suggestions were overseen and participants continued to spell letter by letter (we did not consider this as a false classification). With more experience with the dictionary functionality and letter arrangement, performance may increase. As dictionary integrations and auto-correction methods improve further, usability and efficiency will also increase further.

Another aim of the study was the comparison of low-target and multi-target c-VEP BCI control. The 32-target speller outperformed the 4-target speller significantly in terms of ITR and OCM in the letter-by-letter spelling task. According to the off-line analysis, accuracies of the 4-target speller were considerably higher than the accuracies of the 32-target speller (see Figure 3). Interestingly, however, accuracies in the letter-by-letter spelling task did not differ significantly. While, according to the questionnaire, most participants preferred the 32-target speller, some participants noted that the visual stimulation was overwhelming, especially during the training phase.

Although in this study, a large subject group was tested, it does not reflect the general population due to the low mean age. Previous studies suggest that elderly users achieve lower ITRs^35,36^. Disabled users also tend to achieve lower ITRs^36^. Although successful tests with patients using multi-target systems have been reported^8^, a lower number of targets may be the better option in terms of classification accuracy^22,23,37^. For example, Carvalho et al.^37^ tested SSVEP systems using two, four, and six class layouts with two stroke patients and eight healthy participants. They observed a negative correlation between the number of targets and accuracy reporting 97%, 77%, and 57% for a two, four, and six class interface, respectively. It should be noted that the target size and the number of trials for training differed for the two layouts. Especially the latter difference was significant (24 trials versus 128 trials), which makes a comparison difficult. The off-line accuracies, which are (as expected) better for the 4-target system suggests that much less trials are needed for low-target systems.

The exploratory analysis supports a trend that female participants achieve better accuracies than male participants as observed in several other studies^13,38,39^: Also in the current study, for both the 4-target and the 32-target speller, female users achieved significantly higher accuracies than male users in letter-by-letter spelling.

According to the post-training questionnaires related to the stimulus presentation, participants rated the training for the 32-target speller more exhausting and annoying than for the 4-target speller. This is likely because a much higher number of trials were recorded for the 32-target speller, which made the training much longer. Several methods to reduce or eliminate the training time have been proposed for the f-VEP paradigm: Yuan et al.^6^ generated EEG templates from a large data set from various subjects and transferred it to a new subject. Nakanishi et al.^40^ explored the usage of individual templates in several sessions. Similar approaches could be realized for the c-VEP paradigm.

According to the post-spelling questionnaire related to the stimulus presentation and overall BCI performance, participants rated the 4-target speller as more exhausting, more annoying and slower. These scores reflect the on-line results, which likely impacted the subjective impression. In general, only a few participants rated the flickering sensation during the spelling tasks as exhausting (16% and 5% for the 4-target and 32-target speller) or annoying (14% and 5%). Still, the flickering sensation can be reduced by employing more subtle stimulus patterns^41,42^ or higher flickering rates^43,44^. However, a decrease in performance may be expected as a consequence.

Next to the flickering sensation, another important issue regarding usability is the EEG setup. Using a low number of EEG electrodes/signal channels simplifies the electrode montage. Unfortunately, especially for poor performers, a higher number of EEG channels seems to be required to ensure adequate speed and accuracy. According to our off-line analysis, using 16 instead of 8 electrodes yielded a considerable increase in accuracy for both the 4-target and the 16-target speller. The extension from 16 to 32 electrodes, on the other hand, yielded only a minor increase.

Lastly, another aim of the study was to evaluate the classification model based on individualized filter bank design and multiple correlations. According to the off-line analysis, the methods yielded a substantially higher accuracy, especially for small classification windows. In this study, the filter bank design was based on alpha, beta, and gamma-band related brain activities (i. e. three sub-band components). The weights for the sub-band components were determined individually using the training data. For several participants, the filter bank approach resulted in substantially higher off-line accuracy values in comparison to the standard method. One explanation for this could be that subjects with high alpha activity may yield higher accuracies because the alpha-band activity is less dominant in classification (because of a smaller weight). Chen et al.^20^ have introduced the filter bank approach for the f-VEP paradigm and reported maximal accuracies when using seven sub-band components. For the c-VEP paradigm, a higher number of sub-band decompositions may be applicable as well. It should be noted that additional sub-band components increase the computational complexity significantly. Moreover, to increase the robustness of the non-control state of the asynchronous selection approach, the implementation of pseudo targets which do not trigger a command selection may be applicable. For the f-VEP paradigm, this approach has already been implemented^35,45^, where additional classes, e. g. averages between neighbouring frequencies, were considered during classification to increase overall system robustness. For the c-VEP paradigm, unused bit-shifts could be employed as pseudo targets.

The study explored usability and efficiency of asynchronous BCI speller variations. The dictionary-supported multi-target system yielded higher accuracies than expected and achieved high character output speeds due to the used word suggestion module. While small improvements in terms of EEG-based classification algorithms are still expected and needed, much greater improvements can be made with respect to GUI efficiency and user-friendliness. We encourage researchers to put a greater focus on user-centered features which are currently lacking behind.

## Methods

This section describes the hardware and software setup used in this study; furthermore, details about the subjects and the procedure are provided. The experiment was performed in accordance with the Declaration of Helsinki and approved by the ethical committee of the University Duisburg-Essen, Germany. All participants gave written informed consent before participation and information needed for the analysis were stored anonymously.

### Participants

In total, 58 (29 males, 29 females) able-bodied subjects with mean age of 24.4 years, standard deviation 3.6 years participated. All of them were recruited among students of the Rhine-Waal University of Applied Sciences. They had normal or corrected-to-normal vision, little to no prior experience with BCIs and no experience with the spellers tested. The experiments took approximately 60 minutes. All participants received a financial reward for their participation.

### Hardware

The used computer (Dell Precision 3630 Tower) with operating system Microsoft Windows 10 Education, running on an Intel processor (Intel Core i7-8700K, @3.70 GHz) equipped with 16 GB RAM, and an NVIDIA graphics card (GeForce GTX 1080). The BCI GUI was displayed on a liquid crystal display screen (Acer Predator XB252Q, 1920 $\times$ 1080 pixel, 240 Hz refresh rate).

We used two synchronized EEG amplifiers (g.USBamp, Guger Technologies, Graz, Austria) connected to 32 passive Ag/AgCl signal electrodes according to the international 10/10 system of electrode placement^46^: FCz, C3, C4, CP5, CP3, CP1, CPz, CP2, CP4, CP6, P7, P5, P3, P1, Pz, P2, P4, P6, P8, PO9, PO7, PO3, POz, PO4, PO8, O10, O1, Oz, O2, O9, Iz, O10. The reference electrode was placed at Cz and the ground electrode at AFz. Abrasive electrode gel was applied between the electrodes and the scalp to bring impedances below 5 k$\Omega$. The amplifier setup was the following: a band-pass filter from 2 to 100 Hz and a notch filter around 50 Hz were applied; the sampling frequency, $F_s$, was set to 600 Hz.

### Spellers

**Figure 6:**
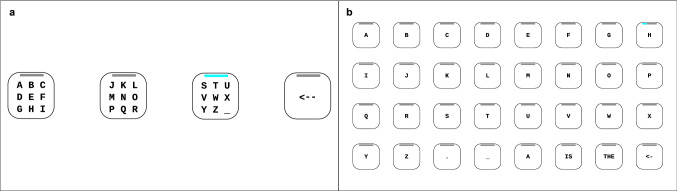
Layouts of the spellers used in the experiment. (**a**) The 4-target speller allowed selection of one of 27 characters in three steps. (**b**) The 32-target speller presented a QWERTZ layout, presenting 28 characters and 3 dictionary suggestions. Both spellers featured an undo option.

Figure 6 shows the user interfaces of the 4-target and the 32-target system. In both spellers, audio (the selected command was voiced) and visual feedback (the selected box increased in size for a short period of time) were provided. Progress bars reflected the current state of the classifier output.

The 4-target speller^13,35^ required three steps to select a letter. Four targets (230 $\times$ 230 pixel) represented the menu options, allowing the selection of 27 characters (26 letters and one underscore/space character) and a correction option. In the first step, the characters were presented into three groups of nine characters each (“A-I”, “J-R”, “S-_”) and the correction option (“$\leftarrow$”) allowed the deletion of the previously selected character. In the second step, the characters of the selected groups were presented in groups of three characters each, and in the third step, individually. In the second and the third step, the correction option (“$\leftarrow$”) allowed the user to go back to the previous step.

To spell the letter “B”, the user had to select the group “A–I” in the first step. The letters were then divided into the sub-groups “A–C”, “D–F”, and “G–I”. After selecting the group “A–C” in the second step, the individual letters “A”, “B” and “C” were presented, and the desired letter “B” could be selected.

The 32-target speller required one step for a selection. The 32 targets (150 $\times$ 150 pixel) represented 28 characters (26 letters, underscore and full stop character), 3 dictionary suggestions, and 1 correction option. The correction option (“$\leftarrow$”) enabled the user to undo the previous selection.

The dictionary suggestions of the 32-target speller were updated after each selection according to an *n*-gram prediction model (as used in our previously developed 8-target speller^27,47^). In general, an *n*-gram model suggests a next item $x_{i}$ for a given sequence of *n* items by considering the probabilities $P(x_i|x_{i-(n-1)},\ldots ,x_{i-1})$. The 32-target interface used a bi-gram ($n=2$), where each item $x_i$ represented a word. The word suggestions were updated according to the previously spelled word.

The prediction model was implemented using a frequency list and a bi-gram list from the Leipzig Corpora Collection^48^, which were based on approximately 1 million English sentences. After each selection, the suggestions were retrieved via structured query language (SQL). We used the database software SQLite to embed the dictionary functionality into our BCI software (written in C++).

To reduce the number of saccades in free communication, the three updated dictionary suggestions (selectable via the corresponding targets in the bottom row) were also displayed as information at the top of a selected target box during the gaze-shifting phase. Thus, the user did not need to move the gaze to the dictionary targets in the bottom row to check if the suggestions were useful. After the gaze-shifting phase, this additional information was removed from the previously selected target.

### Experimental procedure

The study consisted of a session with the 4-target and another session with the 32-target speller. The order of sessions was randomly permuted to reduce the effects of learning and fatigue on the results. Each session consisted of a training phase and a spelling phase. After each phase, a short questionnaire was conducted.

During training, several trials of EEG data were recorded which were used to generate templates for individual c-VEP targets. The training was divided into $n_b$ blocks, where each target of the interface was fixated on for a 2.1 s trial (two full stimulation cycles of the code pattern). For the 32-target speller, $n_b=4$ training blocks were recorded ($N=4\cdot 32 = 128$ trials) and for the 4-target speller, $n_b=6$ training blocks were recorded ($N=6\cdot 4=24$ trials).

The participants initiated the training phase by pressing the space bar. The target they needed to gaze at was highlighted by a green frame. Targets were highlighted from upper left to right and top to bottom. In between trials, the flickering paused for 1 s, and in between blocks, the users could rest.

In the spelling phase a brief familiarization run, where participants learned the functionality of the speller layout was performed. For this, participants went through the copy-spelling tasks “BCI” and “BRAIN”. Thereafter, the pangram “THE QUICK BROWN FOX JUMPS OVER THE LAZY DOG” was spelled (letter-by-letter spelling). Participants were told to spell the phrase letter-by-letter; selections of dictionary suggestions were still possible and treated as false selection. Occurring errors needed to be corrected using the undo functionality. For the 32-target speller, an additional spelling task was performed: Participants typed sentences of varying complexity ($M=36.0$ characters, $SD=5.5$) using the dictionary functionality of the interface (dictionary-supported spelling). Table 1 shows the sentences used for this task.

**Table 1:** Sentences used in the on-line spelling phase with the 32-target speller. Provided are subject number with gender (M/F) and age in years, and the sentence used in the dictionary-assisted spelling task. Each participant had a different spelling task.

Subject	Sentence	Subject	Sentence
1 (M25)	HOW DO YOU LIKE THE PASTA YOU EAT	30 (F20)	THE SCIENCE OF TODAY IS THE TECHNOLOGY OF TOMORROW
2 (M21)	I WANT SOMETHING TO DRINK	31 (F26)	MAN IS THE MEASURE OF ALL THINGS
3 (F19)	COULD YOU PREPARE SOME HOT CHOCOLATE	32 (M24)	WOULD YOU LIKE TO GO NOW OR LATER
4 (M21)	MY MOTHER BAKES REALLY NICE CAKES	33 (M22)	I WOULD LIKE TO COME WITH YOU
5 (M21)	THE WEATHER IN GERMANY IS MOSTLY COLD	34 (F22)	LEARNING IS THE BEGINNING OF WEALTH
6 (F29)	WE WILL MAKE A STUDY ROUND TOMORROW	35 (M34)	ALWAYS GO WHERE THE PEOPLE DANCE
7 (M24)	YOU SPEAK ENGLISH VERY WELL	36 (M27)	DO NOT JUDGE MY PAST I DO NOT LIVE THERE
8 (M21)	WHAT DO YOU DO FOR WORK	37 (F22)	IT IS NEVER GOOD TO BE CRUEL WITH OTHERS
9 (M22)	NEXT TIME WE WILL ORDER THE BIG BURGER	38 (M20)	USE WHAT YOU HAVE AND DO WHAT YOU CAN
10 (M31)	WE WILL GO ON HOLYDAYS NEXT MONTH	39 (F22)	NOTHING REALLY SEEMS TO BE EASY
11 (F25)	I WOULD LIKE TO EAT SOMETHING	40 (F28)	YESTERDAY I FOUND MONEY IN MY PANTS
12 (M21)	ONE APPLE A DAY MAKES THE DOCTOR AWAY	41 (M28)	KNOWING IS NOT ENOUGH WE MUST APPLY
13 (M25)	I TAKE YOGA AND PILATES CLASSES	42 (F21)	YOU ARE NEVER TOO OLD TO SET ANOTHER GOAL
14 (M23)	WOULD YOU MIND IF I GO EARLIER TONIGHT	43 (M24)	YOU SHOULD BE DANCING SALSA NOW
15 (M26)	AT NIGHT OR IN THE MORNING	44 (M21)	WOULD YOU GO TO THE CINEMA WITH ME
16 (M27)	WE LIVE IN THE BEST OF ALL POSSIBLE WORLDS	45 (F20)	SET YOUR GOALS HIGH AND NEVER STOP
17 (M27)	I WOULD LIKE SOME COFFEE	46 (F24)	PROBLEMS ARE NOT STOP SIGNS THEY ARE GUIDELINES
18 (F34)	YOU CANNOT STEP TWICE IN THE SAME RIVER	47 (F23)	IT ALWAYS SEEMS IMPOSSIBLE TILL IT IS DONE
19 (M22)	WHAT ARE YOU GOING TO DO TODAY	48 (F23)	MUSIC IS MOTIVATIONAL AND MAKES YOU RELAX
20 (F24)	LIBERTY CONSISTS IN DOING WHAT ONE DESIRES	49 (M22)	TO SUCCEED YOU MUST FIRST BELIEVE
21 (F29)	I WILL TRY TO MAKE IT RIGHT THIS TIME	50 (F25)	FOLLOW YOUR INNER MOONLIGHT AND DO NOT HIDE
22 (F23)	HOW ABOUT THE KOREAN RESTAURANT	51 (M33)	BE GENTLE WITH ALL AND STERN WITH YOURSELF
23 (M27)	WHAT ARE YOU PLANNING TO DO TODAY	52 (F29)	MAY THE FORCE BE WITH YOU ON MONDAY
24 (F24)	WE MUST TAKE CARE OF OUR PLANET	53 (F23)	SHE LIKES CHOCOLATE ICE CREAM
25 (F24)	PLEASE BRING ALL YOUR DOCUMENTS BY TOMORROW	54 (F23)	THE BANANAS HERE DO NOT TASTE DELICIOUS
26 (F29)	I NEED TO GO TO THE STORE TOO	55 (F19)	THE WINTER TIME IS REALLY COLD AND HUMID
27 (M26)	DO YOU FEEL COMFORTABLE ON THAT CHAIR	56 (F22)	I WILL MAKE HIM AN OFFER HE CAN NOT REFUSE
28 (M22)	I AM ONLY HAPPY WHEN IT RAINS	57 (F25)	MY NAME IS BOND JUST BOND
29 (M28)	HAVING A HEALTHY LIFE IS IMPORTANT	58 (F24)	I WOULD LIKE TO GET SOME SUNSHINE EVERYDAY

### Questionnaires

For both systems, the questionnaires consisted of several questions to assess the subjective impression regarding user-friendliness and efficiency. Two questions were answered after the training phase (post-training questionnaire) and five questions were asked after the spelling task (post-spelling questionnaire). Participants answered on a 7-point Likert scale, where 2 corresponded to complete agreement with a statement, and 7 corresponded to complete agreement with the opposing statement. In this regard, the opposing terms were exhausting versus relaxing, and annoying versus comfortable in the post-training questionnaire, and, in addition, confusing versus clear, boring versus interesting, slow versus fast in the post-spelling questionnaire.

### Stimulus design

Stimulus presentation was realized with circularly shifted 63-bit m-sequences, which have been used in many c-VEP systems^10,11,23^. The stimuli altered between the binary states ’black’ (the background colour, represented by ’0’) and ’white’ (represented by ’1’). The stimulus update rate was set to 60 Hz (a quarter of the monitor refresh rate). The duration of one stimulus cycle was therefore $63/60=1.05\,s$.

The initial code sequence used for the upper left BCI target was defined as

$$\begin{aligned} c_1=101011001101110110100100111000101111001010001100001000001111110. \end{aligned}$$

The remaining stimuli $c_k$, $k=2,\ldots ,K$, were circularly left shifted versions of $c_1$; in this respect, we employed left shifts of $k \cdot 4$ bit and $k \cdot 2$ bit for 4-target and the 32-target speller, respectively.

Stimulus presentation and data acquisition were synchronized via separated timers (one in the stimulus acquisition thread and another one in the stimulus presentation thread)^32^. Time stamps were accessed via system_clock::now from the std::chrono library (the accuracy of the function is hardware dependent).

### Spatial filter design and template generation

We designed spatial filters by conducting CCA on the data collected during the training sessions^23^. Given two multi-dimensional variables $\mathbf{X} \in {\mathbb {R}}^{m_1\times n}$ and $\mathbf{Y} \in {\mathbb {R}}^{m_2 \times n}$, CCA identifies weights $\mathbf{a} \in {\mathbb {R}}^{m_1}$ and $\mathbf{b} \in {\mathbb {R}}^{m_2}$ that maximize the correlation, $\rho$, between the so called canonical variates $\mathbf{x} =\mathbf{X} ^T \mathbf{a}$ and $\mathbf{y} =\mathbf{Y} ^T\mathbf{b}$ by solving

1 $$\begin{aligned} {\rho }=\max _\mathbf{a ,\mathbf{b} } \frac{E[\mathbf{a} ^T \mathbf{X} \mathbf{Y} ^T \mathbf{b} ]}{\sqrt{E[\mathbf{a} ^T \mathbf{X} \mathbf{X} ^T \mathbf{a} ] \, E[\mathbf{b} ^T \mathbf{Y} \mathbf{Y} ^T \mathbf{b} ]}}, \end{aligned}$$

where *E* denotes the expectation operator. The correlation value $\rho$ that solves (1) is the first and also called maximal canonical correlation.

Typically, in VEP research, only this first canonical correlation is used for classification or for the design of spatial filters. However, due to the noisiness of the EEG, information may be distributed over several coefficients^5^. Recently, Mondini et al.^26^ showed that considering multiple correlations can improve signal classification.

CCA identifies further correlations as follows: Determining weights $\mathbf{a} _2$,$\mathbf{b} _2$ maximizing (1) subject to the restriction that the resulting pair of canonical variates is uncorrelated with the first pair yields the second canonical correlation, $\rho _{2}$. These steps can be repeated several times. In general, the number of canonical correlations is equal to the number of rows of the smaller variable. In this respect, CCA yields $m=\min \{m_1,m_2\}$ canonical correlations $\rho _{1}(=\rho ),\rho _{2},\ldots , \rho _{m}$ (sorted from highest to lowest), and the corresponding weight pairs $\mathbf{a} _i,\mathbf{b} _i$, $i=1,\ldots ,m$.

Here, multiple weights were used as well. Each training trial was stored in an $m\times n$ matrix, where *m* denotes the number of electrode channels (here, $m=32$) and *n* denotes the number of sample points (here, two 1.05 s stimulus cycles, $n=1.05 \cdot F_s\cdot 2=1260$). Initially, the *N* recorded trials were circularly shifted to match the phase of the first trial (which corresponded to a bit-shift of 0). The shifted trials $\mathbf{Z} _i\in {\mathbb {R}}^{m\times n}$, $i=1,\ldots ,N$, were then averaged, yielding

2 $$\begin{aligned} \bar{\mathbf{Z }}=\sum _{i=1}^N \frac{1}{N}\mathbf{Z} _i. \end{aligned}$$

From this matrix, templates $\mathbf{X} _i \in {\mathbb {R}}^{m\times n}$, $i=1\ldots K$ for each target class were constructed by circularly shifting, $\bar{\mathbf{Z }}$ according to the bit-shift of the underlying code sequence $c_i$ (see^23^).

Two $m\times N\cdot n$ matrices were constructed to design CCA-based spatial filters,

3 $$\begin{aligned} \tilde{\mathbf{X }}=[\mathbf{Z} _1 \mathbf{Z} _2 \ldots \mathbf{Z} _{N}]\quad \text{ and }\quad \tilde{\mathbf{Y }}=[\underbrace{\bar{\mathbf{Z }}\bar{\mathbf{Z }}\ldots \bar{\mathbf{Z }}}_{\begin{array}{c} N \end{array}}], \end{aligned}$$

applying (1), yields the weight sets $\tilde{\mathbf{a }}_i, \tilde{\mathbf{b }}_i$, $i=1,\ldots ,m$. A subset of the former (the first *s* weights) were used as spatial filters, $\mathbf{w} _i=\tilde{\mathbf{a }}_i$, $i=1,\ldots ,s$. For the presented experiment, the number of considered canonical variates was $s=4$.

### Asynchronous target identification

Every 0.05 s, the classification thread processed received EEG data blocks (stored as $m\times n_a$-matrix, where $n_a=F_s\cdot 0.05=30$). These data blocks were accumulated in a data buffer $\mathbf{Y} \in {\mathbb {R}}^{m\times n_y}$, and compared to reference signals $\mathbf{R} _i \in {\mathbb {R}}^{m\times n_y}$, $i=1,\ldots ,K$, which were constructed as sub-matrices of the templates $\mathbf{X} _i$, containing only the first $n_y$ columns.

Classification was performed for time windows higher or equal to 0.25 s ($n_y\ge 150$) . Correlation values $\lambda _k$, between reference signals and data buffer were calculated as

4 $$\begin{aligned} {\lambda }_k={\rho }\; \left( \begin{bmatrix} \mathbf{Y} ^T \mathbf{w} _1\\ \vdots \\ \mathbf{Y} ^T \mathbf{w} _s \end{bmatrix}, \begin{bmatrix} \mathbf{X _k}^T \mathbf{w} _1\\ \vdots \\ \mathbf{X _k}^T \mathbf{w} _s \end{bmatrix} \right) , \quad k=1,\ldots ,K. \end{aligned}$$

The classification candidate index *C* was determined as

5 $$\begin{aligned} C= \mathop {\hbox {arg max}}\limits _{k=1,\ldots , K} {\lambda }_k \,. \end{aligned}$$

The BCI output associated with *C* was only produced if the distance between the highest and second-highest correlation surpassed a threshold value, $\beta$. Otherwise, further samples were collected. The classification window of length $n_y$ extended incrementally as long as $n_y<n$. When $n_y=n$, the first *n*/2 columns of *Y* were shuffled out. For this window mechanism the samples per data block (here, $n_a=30$) were selected as divider of the cycle length in samples (here, $n/2=630$).

Whenever the threshold criterion was satisfied, the associated BCI output was produced, the data buffer $\mathbf{Y}$ was cleared, and a gaze-shifting period of 1 s followed, where data collection and flickering paused. We used $\beta =0.15$ and $\beta =0.1$ for the 4-target speller and 32-target speller, respectively. These values were determined based on preliminary test runs allowing low time classification windows and high accuracies for the presented layouts. This asynchronous approach was used during on-line tasks; the off-line evaluation in the result section was based on a synchronous approach (i. e. $\beta =0$).

### Individualized filter bank design

The filter banks were generated with an 8th order Butterworth band-pass filter. The three sub-bands used in this study were defined by the following lower and upper cut-off frequencies:

1. the sub-band between 8 and 60 Hz (covering the alpha, beta and gamma-bands),
2. the sub-band between 12 and 60 Hz (covering the beta and gamma bands),
3. the sub-band between 30 and 60 Hz (covering the gamma band).

For each sub-band, a separate set of spatial filters $\mathbf{w} ^{(l)}_i$ and templates $\mathbf{X} _i^{(l)}$ were determined as described before.

For filter bank classification, correlations were calculated for each sub-band independently using (4), which yielded a set of coefficients ${\tilde{\lambda }}^{(j)}_k$, $j=1,2,3$; $k=1,\ldots ,K$. For target identification, the following individualized combination was considered

6 $$\begin{aligned} \lambda _k= a_1{\tilde{\lambda }}_k^{(1)}+a_2{\tilde{\lambda }}_k^{(2)}+a_3{\tilde{\lambda }}_k^{(3)} , \quad k=1,\ldots ,K. \end{aligned}$$

The weights $a_j$, were set to $a_j=\rho ^{(j)}/(\rho ^{(1)}+\rho ^{(2)}+\rho ^{(3)})$, $j=1,2,3$, where $\rho ^{(j)}$ refers for the maximal correlation coefficients obtained via CCA (1) for the respective sub-band decomposition of the matrices in (3). Finally, the class label *C* was again obtained with (5).

### Performance metrics

The classification accuracy, ITR^1^, and OCM^29^ were used to investigate BCI performance.

The classification accuracy, *p*, is calculated as the number of correctly classified selections divided by the total number of selections.

The ITR in bit/min, $B_m$, is calculated as

7 $$\begin{aligned} B_m=\frac{\log _2 K+p\log _2 p+(1-p)\log _2\left( \frac{1-p}{K-1}\right) }{t/60}, \end{aligned}$$

where *K* denotes the number of classes, and *t* denotes the average selection interval (in s). The number of classes was $K=4$ for the 4-target speller and $K=32$ for the 32-target speller. It should be noted, that for the 4-target speller, *K* can be determined by the number of selections in each step (i. e. the number of targets) or by the total number of possible selections (i. e. the number of output characters). In this study, the first option was used, which is applicable to measure the performance with respect to the classification methods (as the classification is performed in each step). An ITR calculation tool can be found at https://bci-lab.hochschule-rhein-waal.de/en/itr.html.

To evaluate speller efficiency, the OCM may be better suited than the ITR. The OCM score is calculated by dividing the number of spelled characters by the spelling time in min (required to complete the entire spelling task). This metric assumes that the user corrects all errors. The metric is applicable to measure the performance with respect to the application efficiency.
